# Skin-Grafting in Loss of Scalp

**Published:** 1881-10-20

**Authors:** William L. Bradley

**Affiliations:** New Haven, Conn.


### SKIN-GRAFTING IN LOSS OF SCALP.

BY WILLIAM L. BRADLEY, M. D., NEW HAVEN, CONN.

In 1869, M. Reverdin, of Paris, France, made known the process of skin-grafting, and it soon became recognized as the best method of healing large granulating surfaces. In cases where the entire scalp has been torn off, the success of this method has attracted especial attention. The following case is believed to be the second of its kind occurring in the United States, and with the first, which was under the care of the late Dr. L. C. Bartlett, of Waterbury, Conn., was partially published in the "Transactions of the Connecticut Medical Society for 1874." In answer to inquiries, and to assist others in the treatment of similar cases, it is now published in completed form.

On the 8th of August, 1873, Ann Farley, aged 35 years, of robust constitution, had her hair caught by a revolving shaft, which tore off the entire scalp. The tear extended below the right eye-brow and down the right cheek; the pinna of the right ear was also removed, and the pericranium covering the vertex was injured to such a degree that, afterward, the outer table of the skull exfoliated for a distance of seven inches. Immediately after the occurrence of the accident the case came under the care of Dr. Ira Smith. Dr. S. replaced the scalp, but in four days was obliged to remove it. The exfoliation of bone was hastened by the application of nitric acid, but more than three months was required for its entire removal. Dr. S. implanted a considerable number of grafts, but, probably on account of their large size, being an inch in diameter, and possibly for other reasons, none of them became permanent.

After nearly four months had elapsed, the patient, who had entered the New Haven Hospital, came under the care of the writer, who continued in charge until the wound was entirely healed. At this time, December 1, 1873, the unhealed surface measured seventy-four square inches. The growth of healthy granulations was so stimulated by the application of basilicon ointment, that, December 10th, several pieces of skin, removed from the patient and others, were successfully implanted. During the following five months the grafting was repeated at varying intervals, but the process of healing was retarded by several attacks of erysipelas.

May 28, 1874, the progress of the case was exhibited to the Annual Convention of the Connecticut Medical Society. At that time the newly formed skin had advanced from one to two inches beyond the usual marginal cicatrization. The denuded surface was entirely healed September 22, 1875, completing a period of two years one and a half month from the time of the accident. This result was accomplished by grafting seven hundred and ninety-five pieces of skin, which measured from a quarter to an eighth of an inch in diameter, and were removed from fifty-five individuals; of this number, one hundred and seventy-one were contributed by a strong and healthy young man, who was resident in the hospital, and one hundred and fifty by the patient. It is estimated that about one-third of the entire number failed to become permanent.

*Constitutional Treatment*—The marked dependence of the condition of the wound upon the state of the general health rendered necessary the continuous employment of supporting measures. The tincture of chloride of iron was used as a blood alterative almost constantly, either to relieve or to ward off the tendency to attacks of erysipelas. The loss of blood attending the monthly flow usually resulted in the destruction of the more recent grafts, and the growth of others which had become permanent was temporarily arrested.

*Condition of the granulating surface.*—Granulations which were small, florid in color, and in the first stage of their growth, were found to possess an adhesive property which was peculiarly favorable to successful grafting. After the first few months the use of stimulating applications was discontinued, because of their tendency to produce large and coarse-textured granulations. Even under the employment of simple cerate, which proved to be the best ordinary dressing, it was found necessary to repress hypertrophy of the granulations by frequent applications of lunar caustic.

*Mode of transplanting.*—The temperature of the apartment in which skin-grafting is to be performed should be about 70° F. In preparation for the reception of grafts, the denuded surface was cleansed with a camel's-hair brush dipped in tepid water; a method of importance, because even a slight effusion of blood was found liable to prevent union between the grafts and the granulations. A piece of skin was seized, not bruised, with a pair of fine-toothed forceps, and, when removed with curved scissors, it measured from a quarter to an eighth of an inch in diameter. The graft was flattened out upon the finger-nail, and both its surfaces were gently scraped to free them from blood, minute hairs, and loose epithelium. Having been pressed down upon, and sometimes between the granulations, the grafts were covered with oiled gutta-percha tissue, surmounted by cotton batting, and supported by a cap of cotton or flannel cloth. As a method for holding the dressing in place, strips of adhesive plaster were found to cause unpleasant pressure, and to impede the circulation of the blood. The number of grafts was only limited by the supply and the extent of the healthy granulating surface. On sixty-six different occasions, an average each time of thirteen grafts were transplanted. In accordance with the observation of others, grafts were found more likely to grow in proportion as they were placed near the margin of the wound, or adjacent to other grafts which had become permanent. The dressings were retained until the third day, and great care was required lest the grafts should be disturbed by the daily dressing of the other parts of the wound. It was observed that grafts which lived and became permanent, but did not grow, retained very closely their original appearance, while others, which did grow, could not be distinguished from the outgrowth of fibro-cellular tissue which surrounded them. When the grafts were placed in near proximity, the newly formed skin appeared to be stronger, and cicatricial contraction was entirely avoided. This was especially noticeable in the treatment of an ectropion of the right upper eye-lid.—*Medical Record.*

